# Revisiting the association of sudden infant death syndrome (SIDS) with polymorphisms of *NHE3* and *IL13*

**DOI:** 10.1007/s00414-023-03139-2

**Published:** 2023-12-13

**Authors:** Dong Qu, Peter Schürmann, Thomas Rothämel, Jessica Fleßner, Daniela Rehberg, Thilo Dörk, Michael Klintschar

**Affiliations:** 1 Institute of Legal Medicine, Hannover Medical School, Carl-Neuberg-Str. 1, 30625 Hannover, Germany; 2 Gynaecology Research Unit, Hannover Medical School, Carl-Neuberg-Str. 1, 30625 Hannover, Germany

**Keywords:** Interleukin 13 (IL13), Sodium/hydrogen exchanger 3 (NHE3/SLC9A3), Sudden infant death syndrome (SIDS), Single-nucleotide polymorphism (SNP)

## Abstract

**Objectives:**

Disturbances of the central nervous system and immune system are thought to play a role in sudden infant death syndrome (SIDS). Dysregulated expression of sodium (Na^+^)/hydrogen (H^+^) exchanger 3 (NHE3) in the brainstem and of interleukin 13 (IL13) in the lungs has been observed in SIDS. An association of single-nucleotide polymorphisms (SNPs) in *NHE3* and *IL13* with SIDS has been proposed, but controversial results were reported. Therefore, there is a need to revisit the association of SNPs in *NHE3* and *IL13* with SIDS.

**Methods:**

Genotyping of rs71597645 (G1131A) and rs2247114 (C2405T) in *NHE3* and rs20541 (+ 4464A/G) in *IL13* was performed in 201 SIDS cases and 338 controls. A meta-analysis was performed after merging our data with previously published data (all from European populations).

**Results:**

Polymorphisms rs2247114 (*NHE3*) and rs20541 (*IL13*) were significantly associated with SIDS overall and in multiple subgroups, but no association was found for rs71597645 (*NHE3*). After combining our data with previously published data, a fixed-effect meta-analysis showed that rs2247114 in *NHE3* retained a significant association with SIDS under a recessive model (OR 2.78, 95%CI 1.53 to 5.06;* p* = 0.0008).

**Conclusion:**

Our findings suggest an association of *NHE3* variant rs2247114 (C2405T), though not rs71597645 (*NHE3*), with SIDS. A potential role of rs20541 (*IL13*) still has to be elucidated. Especially NHE3 seems to be an interesting topic for future SIDS research.

**Supplementary Information:**

The online version contains supplementary material available at 10.1007/s00414-023-03139-2.

## Introduction

Sudden infant death syndrome (SIDS), the leading cause of death of infants in developed countries, refers to the sudden and unexpected death of infants aged younger than 1 year old for that no specific cause of death could be established after autopsy and death scene investigation [1]. The triple-risk hypothesis was put forward to aid in interpreting the role of interconnected risk factors of SIDS such as vulnerable infants (e.g., genetic impact and preterm birth), critical developmental period (e.g., 2 to 4 months), and extrinsic factors (e.g., infection and co-sleeping). Nevertheless, the concrete pathogenesis of SIDS is still unknown [2].

Among other hypotheses, it has been proposed that SIDS might at least in part be attributed to a disturbed homeostasis in the central nervous system (CNS). Neurotransmitter imbalances in brainstem and hypothalamic areas that are involved in the control of cardiovascular and respiratory functions and might trigger SIDS by induced respiratory or cardiac arrest, have been associated with SIDS [3–5]. The sodium (Na^+^)/hydrogen (H^+^) exchanger 3 (NHE3), as a vital Na^+^/H^+^ antiporter, was reported to be highly expressed in the brainstem of SIDS, which might lead to altered breathing control and subsequently SIDS [6]. Moreover, the same research group identified three single-nucleotide polymorphisms (SNPs) from *NHE3* (missense variant: C2405T; promoter variants: G1131A and C1197T) that were significantly associated with SIDS [7] and might explain the previously reported overexpression of NHE3 in the brainstem. However, a subsequent study failed to confirm these results [8].

Another hypothesis suggests an imbalanced immune response, e.g., hyper- or hypo-inflammatory reactions and hypersensitivity, might contribute to SIDS [9–14]. Interleukin 13 (IL13) as a T helper 2 (Th2) cell-related inflammatory mediator was linked to anti-inflammation response and allergic inflammation [15]. We previously reported that IL13 was decreased in the lung lysate of SIDS, but increased in the thymus, suggesting that IL13 might be involved in a proposed impaired immune status of SIDS [16, 17]. Moreover, one Norwegian study investigating the role of cytokine SNPs in SIDS found that the missense polymorphism *IL13* + 4464 (A/G) was not relevant to SIDS, but its genotype GG was associated with infectious infant deaths [18]. Interestingly, *IL13* + 4464 (rs20541) was suggested to impact the expression of IL13 [19]. Thus, we argue that the *IL13* + 4464 polymorphism might be linked to the observed differently expressed IL13 levels in SIDS and thus be a polymorphism of interest in SIDS.

As mentioned above, several studies in SIDS with partially inconsistent findings investigated potential abnormalities of NHE3 and IL13. We argue that further data are needed to be able to corroborate or refute a role in the etiology of SIDS. Therefore, we hypothesized that revisiting the potential associations between SIDS and SNPs from *IL13* and *NHE3* in an independent case–control study might aid in understanding possible mechanisms of SIDS from a genetic viewpoint. To this end, genotyping of three SNPs in *NHE3* and *IL13* was performed in an independent cohort from Hannover, and a meta-analysis combined with other published results was carried out.

## Materials and methods

### Samples

Samples from 201 SIDS and 338 control cases from Lower Saxony, Germany, were collected at the Institute of Legal Medicine, Hannover Medical School, between 2003 and 2020 (with the exception of 2 cases from 1989). The enrollment criteria of the SIDS cohort (*n* = 201) conformed to the San Diego definition of SIDS. The SIDS cases in this study have not been previously shared with the other SIDS-related papers included in the meta-analysis. The sex ratio (male:female) in the SIDS group is 58.7%:41.3%. The age range of SIDS cases was 5 ~ 342 days, with an average of 131 days. The control cohort (*n* = 338) was comprised of 305 adults who had survived from the risk age range of SIDS and 33 infants died of specific causes rather than SIDS within the 1st year of their life. The local ethics committee of Hannover Medical School approved this study.

### Candidate SNPs and genotyping

The rsID number of three reported *NHE3* SNPs was confirmed based on the primer sequence provided in the article by Poetsch et al. [7] using the Basic Local Alignment Search Tool (BLAST) (https://blast.ncbi.nlm.nih.gov/Blast.cgi). The rsID numbers were rs2247114 (C2405T), rs71597645 (G1131A), and rs187829972 (C1197T). Minor allele frequency (MAF) data of the SNPs in the European population of the 1000G database were compared to the corresponding MAF calculated from the published data. Variant rs187829972 (C1197T) had a MAF lower than 0.05 in the European population (0.003) so we had insufficient power for this variant in our study. Thus, only rs2247114 (C2405T) and rs71597645 (G1131A) were included for *NHE3*. *NHE3* rs2247114 (A/G) in this study and *NHE3* C2405T in previous studies describe the same variant, but the effect allele is termed A in the present study to conform with NCBI reference nomenclature. In the *IL13* gene, the rsID number of *IL13* + 4464 (A/G) was mentioned to be rs20541 by Ferrante et al. [18].

Genomic DNA was isolated from the SIDS cases and controls following the manufacturer’s instructions for the QIAamp DNA Mini Kit (Qiagen, Hilden, Germany). Details of the genotyping procedure using 192.24 Genotyping Dynamic Arrays and the Biomark EP1 platform were described in our previous article [20]. The detailed information of probes and primers are listed in Supplementary Material S1.

### Statistical analysis

Hardy–Weinberg equilibrium (HWE) in controls was checked using an online HWE calculator (https://wpcalc.com/en/equilibrium-hardy-weinberg/). A 2 × 2 Fisher’s exact test was employed to test the association of SNPs and SIDS using dominant as well as recessive models, and a linear-by-linear model of the χ^2^ test was used under the additive model. Odds ratios (ORs), 95% confidence intervals (CIs), and corresponding *p* values were calculated. In order to achieve a combined analysis of our data and published data, the statistical analysis of alleles/genotypes was unified and re-performed based on the genotype distribution of published results. A fixed (common)-effects meta-analysis with Mantel–Haenszel odds ratios was performed using the meta package (version 6.5–0) in R (version 4.3.0). Forest plots of all the fixed-effects meta-analyses are given in Supplementary Material S2.

To estimate the possible functional relevance of gene expression, cis-eQTL target gene expression was checked using online data from the Genotype-Tissue Expression (GTEx) project (https://www.gtexportal.org/home/).

In all statistical analyses, a two-sided *p* value < 0.05 was considered to indicate statistical significance. All statistical analyses were performed using SPSS 24.0 software (SPSS Inc. Chicago, IL, USA) or R (version 4.3.0).

## Results

In this study, a total of 539 samples (SIDS = 201, controls = 338) were successfully genotyped for 3 SNPs, having a 99.32% call rate and a 96.77% concordance rate within 31 replications. All results were in accordance with Hardy–Weinberg expectations.

### NHE3

Regarding rs71597645 (G1131A), no significant association with SIDS was found in the genotyping data of either this study or in the combined analysis (Table 1). Also, no significant findings were observed after the stratified analyses of this study.

**Table 1:** Analysis on the association of rs71597645 (G1131A) and rs2247114 (C2405T) in *NHE3* with SIDS

SNP	Genotypes/Alleles or *p* value		This study		Studer *et al.*		Poetsch *et al.*		Meta-analysis	
			Control	SIDS	Control	SIDS	Control^#^	SIDS	Control	SIDS
rs71597645 (G1131A)	GG		230	137	129	113	168	168	527	418
	GA		91	56	60	44	52	75	203	175
	AA		17	5	3	3	0	8	20	16
	*p* value&OR (95%CI)*	Dominant (AA/GA *vs.* GG)	*p*: 0.85, 0.95 (0.65−1.38)		*p*: 0.56, 0.85 (0.54−1.34)		***p*** **: 0.03, 1.60 (1.06−2.40)**		*p*: 0.43, 1.10 (0.87−1.39)	
		Recessive (AA *vs.* GA/GG)	*p*: 0.18, 0.49 (0.18−1.35)		*p*: 0.99, 1.20 (0.24−6.05)		***p*** **: 0.008, 15.39 (0.88−268.30)†**		*p*: 0.66, 1.11 (0.56−2.20)	
	G		551	330	318	270	388	411	1257	1011
	A		125	66	66	50	52	91	243	207
	*p* value&OR (95%CI)*		*p*: 0.51, 0.88 (0.64−1.22)		*p*: 0.61, 0.89 (0.60−1.33)		***p*** **: 0.008, 1.65 (1.14−2.39)**		*p*: 0.43, 1.09 (0.88−1.34)	
rs2247114 (C2405T)	GG		257	166	132	118	183	199	572	483
	GA		71	24	58	40	28	25	157	89
	AA		5	10	2	2	9	27	16	39
	*p* value&OR (95%CI)*	Dominant (AA/GA *vs.* GG)	*p*: 0.12, 0.69 (0.44−1.09)		*p*: 0.35, 0.78 (0.49−1.25)		*p*: 0.29, 1.29 (0.81−2.06)		*p*: 0.35, 0.88 (0.68−1.15)	
		Recessive (AA *vs.* GA/GG)	***p*** **: 0.03, 3.45 (1.16−10.25)**		*p*: 0.99, 1.20 (0.17−8.63)		***p*** **: 0.008, 2.83 (1.30−6.15)**		***p*** **: 0.0008, 2.78 (1.53−5.06)**	
	G		585	356	322	276	394	423	1301	1055
	A		81	44	62	44	46	79	189	167
	*p* value&OR (95%CI)*		*p*: 0.62, 0.89 (0.60−1.32)		*p*: 0.40, 0.83 (0.54−1.26)		***p*** **: 0.02, 1.60 (1.08−2.36)**		*p*: 0.54, 1.07 (0.86−1.35)	

Note: The control^#^ cohort was composed of infant and adult samples. The *p* value and OR (95%CI)* were re-calculated according to the original genotype/allele distribution data. The OR (95%CI)† values were calculated by the Woolf logit method as one cell value is 0. *SIDS* Sudden infant death syndrome; *OR* Odds ratio; *CI* Confident interval

**Fig. 1:**
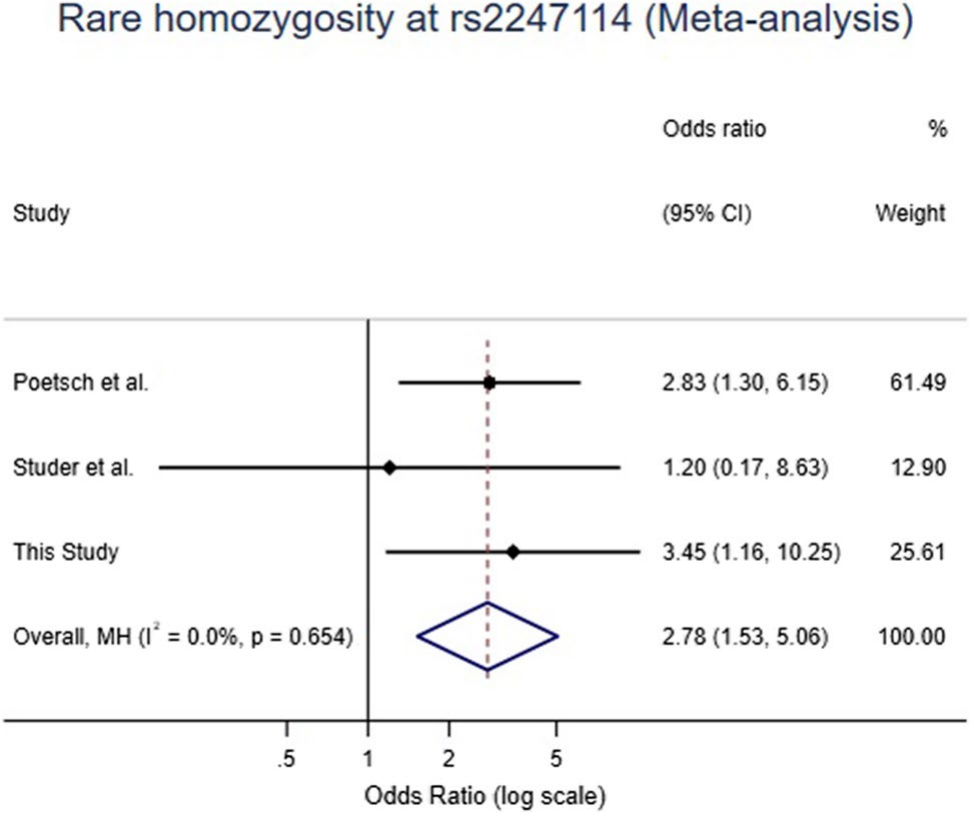
Forest plot from a fixed-effect meta-analysis of *NHE3* rs2247114 (C2405T) genotype AA in SIDS cases and controls in combination with available case–control studies. OR: Mantel–Haenszel odds ratio from fixed-effects meta-analyses. CI: confidence interval. *I*-square and *p* values characterize the degree of heterogeneity among studies

For rs2247114 (C2405T), an association of this locus with SIDS was unveiled under the recessive model (risk genotype: AA, *p* = 0.03, OR = 3.45, 95%CI 1.16–10.25) (Table 1). The strength of this association was further increased after merging data from this study with previously published ones (risk genotype: AA, *p* = 0.0008, OR = 2.78, 95%CI 1.53–5.06) (Table 1 and Fig. 1). For the stratified analysis in this study (Table 3), genotype AA of rs2247114 was found to be also associated with increased risk in SIDS subgroups such as male infants, infants aged 4–8 months, and deaths occurring in autumn and winter.

### IL13

With regard to rs20541 (*IL13* + 4464), genotypes AA/GA were found to be associated with SIDS at the overall level (*p* = 0.04; OR = 0.68, 95%CI 0.47–0.97) (Table 2). However, statistical significance was no longer reached after pooling our data with previously published ones (Table 2). In the stratified analysis of this study (Table 3), rs20541 was associated with risk in multiple SIDS subgroups (e.g., female, age 0–4 months, age 2–4 months, and non-prone sleeping positions).

**Table 2:** Analysis on the association between rs20541 in *IL13* and SIDS

Genotypes/Alleles or *p* value		This study		Ferrante *et al.*		Meta-analysis	
		Control	SIDS	Control	SIDS^#^	Control	SIDS
GG		195	134	80	113	275	247
GA		127	55	46	78	173	133
AA		15	11	5	9	20	20
*p* value&OR (95%CI)*	Dominant (AA/GA *vs.* GG)	***p*** **: 0.04, 0.68 (0.47−0.97)**		*p*: 0.43, 1.21 (0.78−1.90)		*p*: 0.27, 0.85 (0.64−3.13)	
	Recessive (AA *vs.* GA/GG)	*p*: 0.68, 1.25 (0.56−2.78)		*p*: 0.99, 1.19 (0.39−3.63)		*p*: 0.54, 1.23 (0.64−2.35)	
G		517	323	206	304	723	627
A		157	77	56	96	213	173
*p* value&OR (95%CI)*		*p*: 0.13, 0.79 (0.58−1.07)		*p*: 0.45, 1.16 (0.80−1.70)		*p*: 0.48, 0.92 (0.73−1.16)	

Note: The SIDS^#^ cohort was composed of SIDS and boardline SIDS cases. The *p* value and OR (95%CI)* were re-calculated according to the original genotype/allele distribution data. *SIDS* Sudden infant death syndrome; *OR* Odds ratio; *CI* Confident interval

**Table 3:** Selected associations of polymorphisms with SIDS subgroups

Stratum	Gene	SNP	Genotype distribution	Additive (Y vs. X alleles)		Dominant (YY/XY vs. XX genotypes)		Recessive (YY vs. XX/XY genotypes)	
			Case no. in SIDS (XX:XY:YY)	OR (95%CI)*	*p* value*	OR (95%CI)	*p* value	OR (95%CI)	*p* value
Male	*NHE3*	rs2247114	96:14:08	1.052 (0.663 ~ 1.630)	0.835	0.775 (0.457 ~ 1.325)	0.344	**4.594 (1.539 ~ 13.72)**	**0.007**
Age 4–8 months		rs2247114	32:02:03	0.875 (0.416 ~ 1.827)	0.852	0.528 (0.217 ~ 1.355)	0.293	**6.059 (1.516 ~ 24.21)**	**0.036**
Autumn + winter		rs2247114	71:09:05	0.910 (0.539 ~ 1.529)	0.792	0.667 (0.364 ~ 1.250)	0.238	**4.081 (1.221 ~ 13.64)**	**0.033**
Female	*IL13*	rs20541	60:21:01	**0.521 (0.319 ~ 0.848)**	**0.008**	**0.504 (0.295·0.859)**	**0.011**	0.265 (0.035 ~ 2.037)	0.295
Age 0–4 months		rs20541	74:22:06	**1.519 (1.007 ~ 2.292)**	**0.045**	**0.520 (0.320 ~ 0.844)**	**0.008**	1.342 (0.507 ~ 3.559)	0.553
Age 2–4 months		rs20541	52:18:04	1.436 (0.902 ~ 2.286)	0.126	**0.581 (0.337 ~ 1.000)**	**0.048**	1.227 (0.395 ~ 3.802)	0.723
Other sleep positions		rs20541	13:2:0	**4.478 (1.037 ~ 19.324)**	**0.029**	**0.211 (0.047 ~ 0.951)**	**0.026**	0.521 (0.028 ~ 9.843)†	0.856

*p* value < 0.05 marked in bold. OR* and *p* values* in the additive model were calculated using the linear-by-linear association of the chi-square test. Alleles X and Y represent major and minor alleles, respectively. OR values marked with † were the Woolf logit method when an empty cell existing in chi-square tables

## Discussion

In this study, two variants of *NHE3* and one of *IL13* were genotyped in 201 SIDS and 338 control cases to further investigate previously reported associations with SIDS. Statistical analyses after genotyping showed evidence for two positive associations with risk (*NHE3*, rs2247114 (C2405T), AA, *p* = 0.03; *IL13*, rs20541 (*IL13* + 4464), AA/GA, *p* = 0.04) while *NHE3* rs71597645 (G1131A) was not associated at the overall level. In the stratified analysis, the two significant variants (rs2247114 (C2405T) from *NHE3* and rs20541 (*IL13* + 4464) from *IL13*) were also linked to multiple subgroups of SIDS. In regard to multiple testing, these results would not remain formally significant after Bonferroni correction, but it is noteworthy that two out of three previously reported variants were replicated. We performed a meta-analysis with the data from this study and previous articles and found that the *NHE3* variant rs2247114 (C2405T) reached statistical significance at a level of *p* < 0.001, lending strong support to its role in SIDS susceptibility.

### NHE3

NHE3 plays a vital role in maintaining adequate respiratory function, and increased levels of NHE3 in the brainstem of rabbits cause maladaptive hyperventilation [21]. NHE3 is one of several mechanisms involved in the water transport that might be involved in the etiology of SIDS, others being, e.g., aquaporins and sulfonylurea receptor 1 (SUR1)-transient receptor potential melastatin 4 (TRPM4) [20, 22]. In the brainstem of SIDS, increased NHE3 expression was detected [6], which might lead to respiratory maladaptation and subsequently trigger SIDS. Three SNPs (rs71597645 (G1131A), rs2247114 (C2405T), and rs187829972 (C1197T)) that may explain the overexpression of NHE3 were reported to be associated with SIDS [7]. However, conflicting results showing a lack of association of these three variants with SIDS were reported in a validation study [8]. In our study, no significant association of rs71597645 with SIDS was observed, even after meta-analyses. However, the genotype AA of rs2247114 (C2405T) was observed to be more frequent in SIDS (SIDS: 5.0%, controls: 1.5%, *p* = 0.03), which is in line with results reported by Poetsch et al. (*p* = 0.008; OR 2.83, 95%CI 1.30–6.15; SIDS: 10.8%, controls: 4.1%) [7]. The only other study in this context reported nonsignificant findings that, however, showed a trend in the same direction (SIDS: 1.3%, controls: 1.0%) [8]. As explained above, the nomenclature used by these earlier studies differs from ours as we typed the different strands, meaning that allele T in the other studies corresponds to allele A in ours. Interestingly, after a meta-analysis using all three datasets, genotype AA of SNP rs2247114 (C2405T) was highly associated with SIDS (*p* = 0.0008, SIDS: 6.4%, controls: 2.1%), suggesting that NHE3 might indeed be involved in the etiology of SIDS.

When the previous studies by Poetsch et al. [7] and Studer et al. [8] were published, no data on the possible functional consequences of these SNPs were available. At present, the question of the biological function of rs2247114 (C2405T) is still not finally resolved: One in vitro study reported that allele A of rs2247114 (C2405T) causes a lower expression of NHE3 [23]. However, another study argued that this lower total NHE3 expression indeed existed, but higher surface expression and increased sensitivity to ligands of NHE3 might compensate for this effect [24]. In human tissues, based on the results from the GTEx database, the genotype AA of rs2247114 (C2405T) is linked to a lower NHE3 expression in multiple brain subregions (the same is valid for other *NHE3* SNPs highly linked (*R*^2^ > 0.8) to rs2247114). Thus, it is difficult to reconcile the enrichment of the AA genotype in SIDS with the increased NHE3 expression in the brainstem of SIDS [6]. As SIDS is regarded as a polygenic disease, NHE3 overexpression in SIDS might be the consequence of other genetic variants (from *NHE3* or other genes) or the consequence of other mechanisms, e.g., as part of a network of seriously imbalanced neurotransmitters, with some decreased and others elevated. Nevertheless, our findings further emphasize that NHE3 might be an important factor in the etiology of SIDS, whose specific functional role should be studied in depth.

### IL13

As mediators of the immune system, cytokines play a vital role in regulating inflammatory responses against infections that are reckoned as one of the underlying risk factors for SIDS. Several studies investigating the role of infections and the immune status in SIDS showed diverse findings on cytokines at gene and gene expression levels [14, 16, 25–28].

Among other cytokines, IL13, one of the Th2-related cytokines, and the gene coding for it have been studied in cytokine-related SIDS studies [16–18]. It has been reported that the pulmonary IL13 level is decreased in SIDS, which seems to suggest a locally impaired immune status [16]. From the genetic point of view, the *IL13* polymorphism rs20541 (*IL13* + 4464) has been linked to altered plasma IL13 levels [19]. However, in the only prior study on SIDS, this variant has not been associated with SIDS [18]. Nevertheless, we postulated that rs20541 might nevertheless participate in the complicated and potentially impaired immunological process in SIDS and decided to re-assess the association of rs20541 with SIDS in our independent SIDS cohort.

Genotypes AA/GA of *IL13* rs20541 were found to be less frequent in SIDS (*p* = 0.04, SIDS: 33.0%, control: 42.1%). This variant is of some impact on the immune system, as it was reported to be associated not only with elevated IL13 levels [19] but also IgE levels [29] and lymphocyte counts [30]. Thus, a decreased proportion of rs20541 genotypes AA/GA in SIDS might be associated with the altered IL13 level observed earlier by our group [16, 17]. However, as no significant results were retained after meta-analyses, we propose no substantial or, at best, a weak association of *IL13* SNP rs20541 with SIDS.

## Conclusion

In summary, this study re-evaluated the association between SIDS and three SNPs from *NHE3* and *IL13* previously reported to be associated with SIDS. In our study, this association could be replicated for two of these three SNPs (rs2247114 in *NHE3* and rs20541 in *IL13*). However, after combining published data with our data, only the *NHE3* SNP rs2247114 remained associated with SIDS. This association, however, was strong (*p* = 0.0008). NHE3 is reported to be overexpressed in SIDS, though the AA genotype that we found accumulated in SIDS seems to be rather associated with a lower expression of NHE3 than with an overexpression. Although our data thus further corroborate that NHE3 might be an important risk factor for SIDS, the specific role of NHE3 overexpression and rs2247114 requires further investigation. Furthermore, our study underlines that the validity of gene-association studies greatly depends on sample size and replication studies to allow for meta-analyses on published suspected gene variants.

### Supplementary Information

Below is the link to the electronic supplementary material.

Supplementary file1 (XLSX 88 KB)

Supplementary file2 (PDF 620 KB)

## References

[1] Krous HF, Beckwith JB, Byard RW (2004). Sudden infant death syndrome and unclassified sudden infant deaths: a definitional and diagnostic approach. Pediatrics.

[2] Guntheroth WG, Spiers PS (2002). The triple risk hypotheses in sudden infant death syndrome. Pediatrics.

[3] Hunt CE (1992). The cardiorespiratory control hypothesis for sudden infant death syndrome. Clin Perinatol.

[4] Kahn A, Groswasser J, Rebuffat E (1992). Sleep and cardiorespiratory characteristics of infant victims of sudden death: a prospective case-control study. Sleep.

[5] Schechtman VL, Harper RM, Kluge KA, Wilson AJ, Southall DP (1990). Correlations between cardiorespiratory measures in normal infants and victims of sudden infant death syndrome. Sleep.

[6] Wiemann M, Frede S, Tschentscher F (2008). NHE3 in the human brainstem: implication for the pathogenesis of the sudden infant death syndrome (SIDS)?. Adv Exp Med Biol.

[7] Poetsch M, Nottebaum BJ, Wingenfeld L, Frede S, Vennemann M, Bajanowski T (2010). Impact of sodium/proton exchanger 3 gene variants on sudden infant death syndrome. J Pediatr.

[8] Studer J, Bartsch C, Haas C (2014). Sodium/proton exchanger 3 (NHE3) and sudden infant death syndrome (SIDS). Int J Legal Med.

[9] Blood-Siegfried J (2009). The role of infection and inflammation in sudden infant death syndrome. Immunopharmacol Immunotoxicol.

[10] Opdal SH (2018) Cytokines, infection, and immunity. In: Duncan JR, Byard RW (eds). SIDS sudden infant and early childhood death: the past, the present and the future. Adelaide (AU): University of Adelaide Press 30024688

[11] Ferrante L, Rognum TO, Vege Å, Nygård S, Opdal SH (2016). Altered gene expression and possible immunodeficiency in cases of sudden infant death syndrome. Pediatr Res.

[12] Buckley MG, Variend S, Walls AF (2001). Elevated serum concentrations of beta-tryptase, but not alpha-tryptase, in sudden infant death syndrome (SIDS). An investigation of anaphylactic mechanisms. Clin Exp Allergy : J Br Soc Allergy Clin Immunol.

[13] Hafke A, Schürmann P, Rothämel T, Dörk T, Klintschar M (2019) Evidence for an association of interferon gene variants with sudden infant death syndrome. Int J Legal Med 133:863–9. 10.1007/s00414-018-1974-6 10.1007/s00414-018-1974-6 30617847

[14] Fard D, Läer K, Rothämel T et al (2016) Candidate gene variants of the immune system and sudden infant death syndrome. Int J Legal Med 130:1025–33. 10.1007/s00414-016-1347-y 10.1007/s00414-016-1347-y 26975745

[15] de Vries JE (1998). The role of IL-13 and its receptor in allergy and inflammatory responses. J Allergy Clin Immunol.

[16] Qu D, Engelmann TA, Preuss V (2023). Pulmonary immune profiling of SIDS: impaired immune maturation and age-related cytokine imbalance. Pediatr Res.

[17] Qu D, Preuss V, Hagemeier L (2023). Age-related cytokine imbalance in the thymus in sudden infant death syndrome (SIDS). Pediatr Res.

[18] Ferrante L, Opdal SH, Vege A, Rognum T (2010). Cytokine gene polymorphisms and sudden infant death syndrome. Acta Paediatr (Oslo, Norway : 1992).

[19] Abdulla AA, Shaheed Mahmood N (2022). Correlation between IL-13rs20541(A> G) gene polymorphism and bronchial asthma among Iraqi patients. Rep Biochem Mol Biol.

[20] Qu D, Schürmann P, Rothämel T, Dörk T, Klintschar M (2022) Variants in genes encoding the SUR1-TRPM4 non-selective cation channel and sudden infant death syndrome (SIDS): potentially increased risk for cerebral edema. Int J Legal Med 136:1113–20. 10.1007/s00414-022-02819-9 10.1007/s00414-022-02819-9 PMC9170623 35474489

[21] Wiemann M, Frede S, Bingmann D, Kiwull P, Kiwull-Schöne H (2005). Sodium/proton exchanger 3 in the medulla oblongata and set point of breathing control. Am J Respir Crit Care Med.

[22] Opdal SH, Ferrante L (2021) Aquaporin-1 and aquaporin-9 gene variations in sudden infant death syndrome. Int J Legal Med 135:719–25. 10.1007/s00414-020-02493-9 10.1007/s00414-020-02493-9 PMC8036210 33462668

[23] Zhu XC, Sarker R, Horton JR (2015). Nonsynonymous single nucleotide polymorphisms of NHE3 differentially decrease NHE3 transporter activity. Am J Physiol Cell Physiol.

[24] Yin J, Tse CM, Cha B (2017). A common NHE3 single-nucleotide polymorphism has normal function and sensitivity to regulatory ligands. Am J Physiol Gastrointest Liver Physiol.

[25] Courts C, Madea B (2011). No association of IL-10 promoter SNP -592 and -1082 and SIDS. Forensic Sci Int.

[26] Ferrante L, Opdal SH, Vege A, Rognum TO (2008). TNF-alpha promoter polymorphisms in sudden infant death. Hum Immunol.

[27] Vennemann MM, Loddenkötter B, Fracasso T (2012). Cytokines and sudden infant death. Int J Legal Med.

[28] Rognum IJ, Haynes RL, Vege A, Yang M, Rognum TO, Kinney HC (2009). Interleukin-6 and the serotonergic system of the medulla oblongata in the sudden infant death syndrome. Acta Neuropathol.

[29] Granada M, Wilk JB, Tuzova M (2012). A genome-wide association study of plasma total IgE concentrations in the Framingham Heart Study. J Allergy Clin Immunol.

[30] Vuckovic D, Bao EL, Akbari P (2020). The polygenic and monogenic basis of blood traits and diseases. Cell.

