# Supplementary figures and images for: Revisiting the association of sudden infant death syndrome (SIDS) with polymorphisms of NHE3 and IL13
Source: Int J Legal Med. 2023 Dec 13;138(3):743–9. doi: 10.1007/s00414-023-03139-2 (PMC11003888; doi:10.1007/s00414-023-03139-2)

# Supplementary Material S2

## NHE3 rs71597645 (A/G)

AA/GA

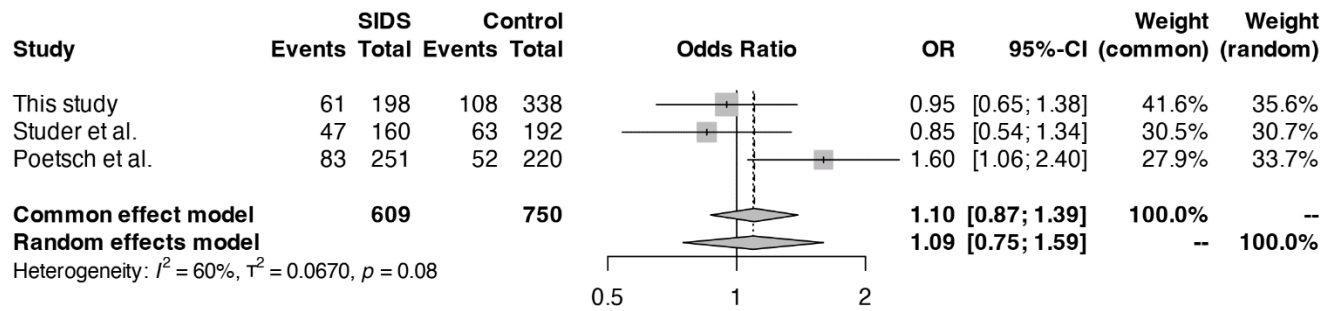

AA

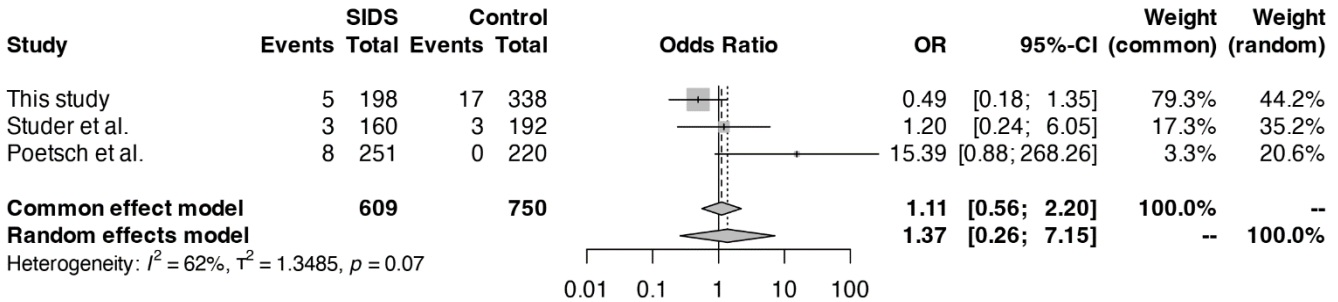

A

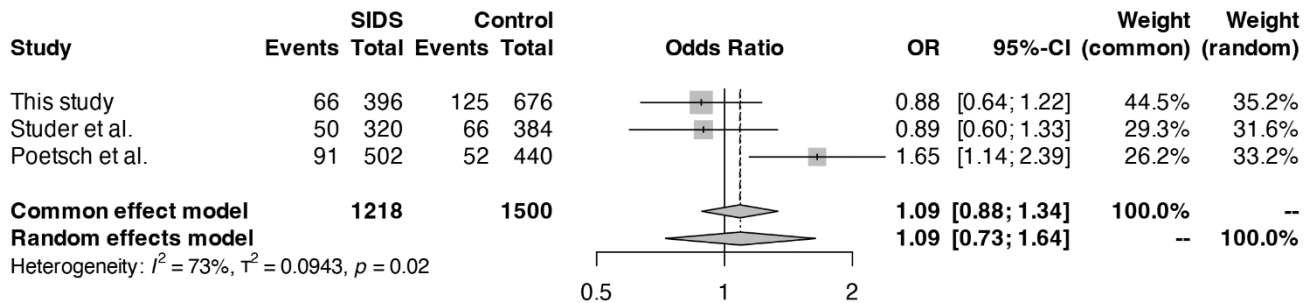

## NHE3 rs2247114 (A/G)

AA/GA

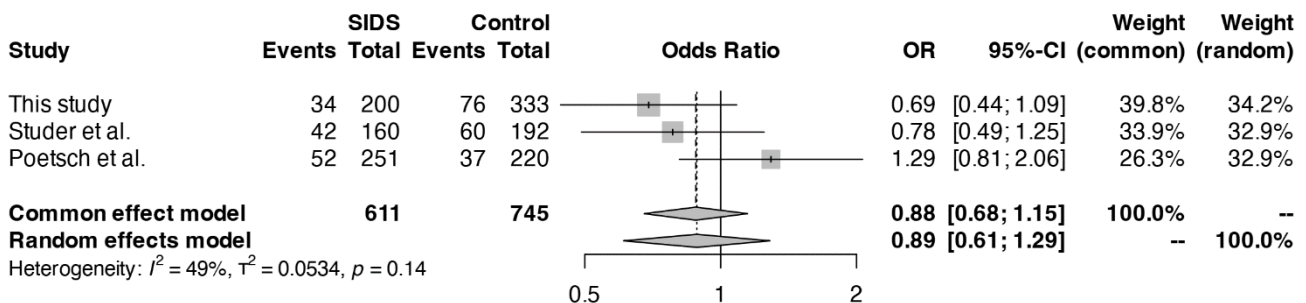

AA

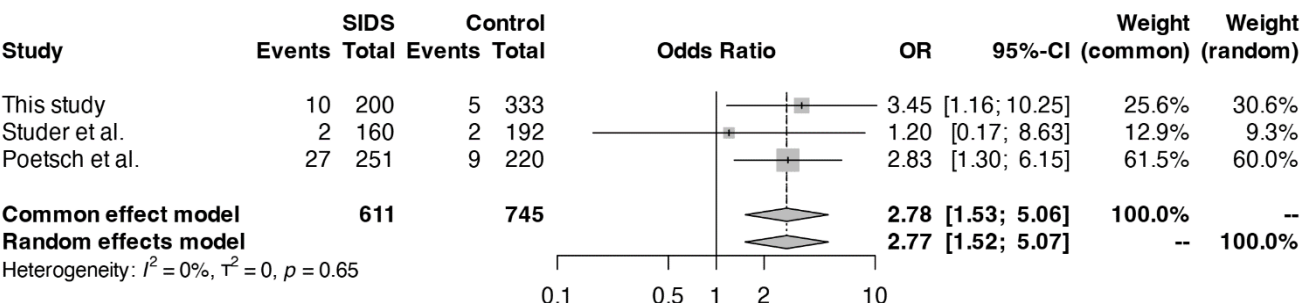

A

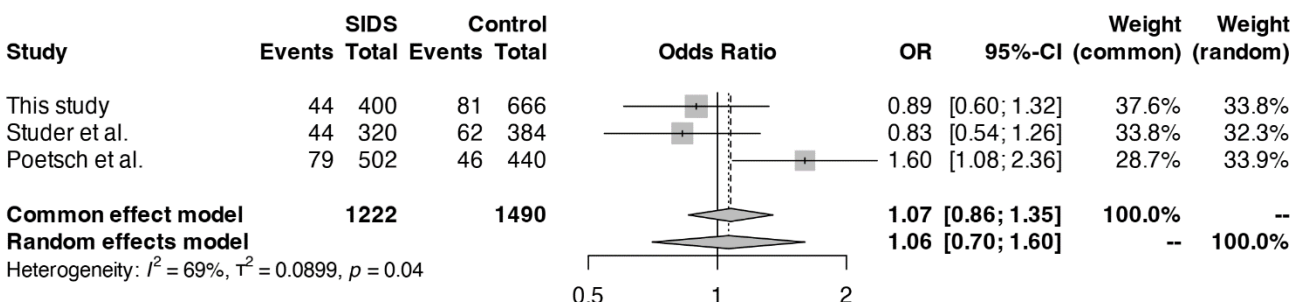

IL13 rs20541 (A/G)

AA/GA

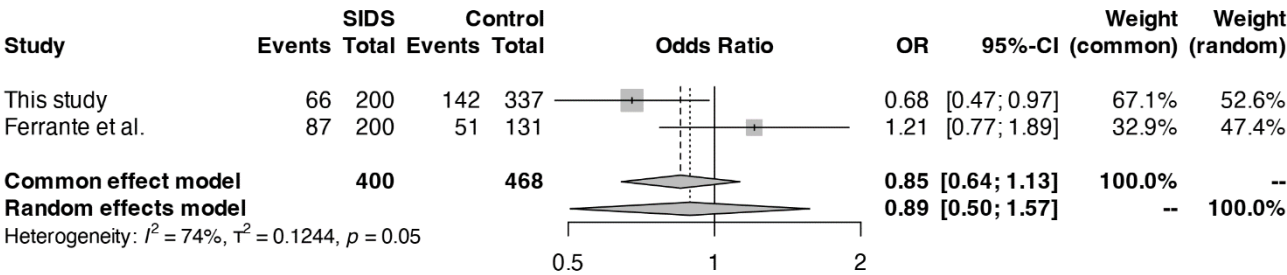

AA

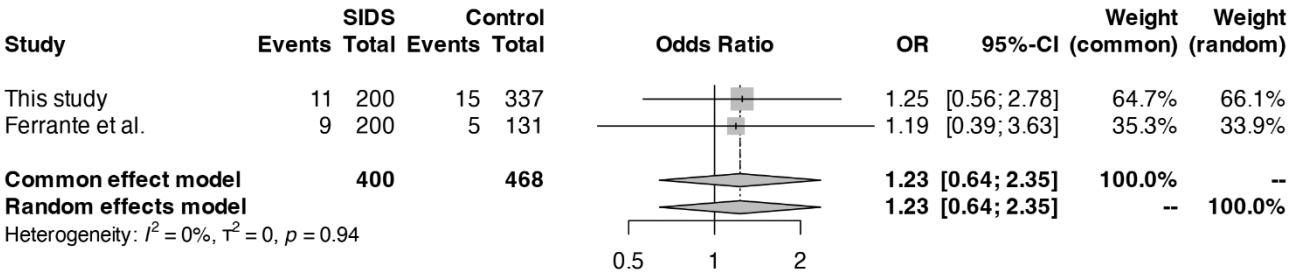

A

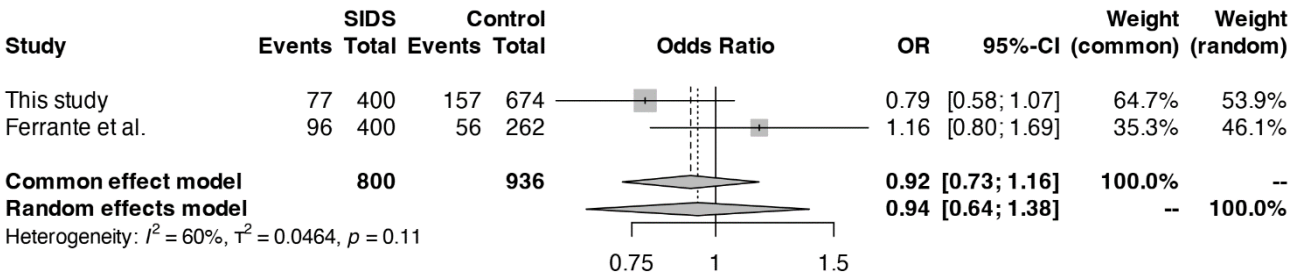

Supplement: Supplementary file 2 — Supplementary file2 (PDF 620 KB) [file 414_2023_3139_MOESM2_ESM.pdf]
